# Correction: Determinants of the use of modern contraceptives among women of reproductive age group in Ethiopia: A multilevel mixed effects analysis

**DOI:** 10.1371/journal.pone.0308582

**Published:** 2024-08-05

**Authors:** Molalign Gualu Gobena, Maru Zewdu Kassie

There is an error in the affiliation for authors Molalign Gualu Gobena and Maru Zewdu Kassie. The correct affiliation is: Department of Statistics, Assosa University, Assosa, Benishangul-Gumuz Regional State, Ethiopia.
