# Supplementary material for: Determinants of the use of modern contraceptives among women of reproductive age group in Ethiopia: A multi-level mixed effects analysis
Source: PLoS One. 2024 Jul 5;19(7):e0306635. doi: 10.1371/journal.pone.0306635 (PMC11226071; doi:10.1371/journal.pone.0306635)
Supplement: Attachment — Submitted filename: Comments.docx [file pone.0306635.s002.docx]

Dear Editor, Thank you very much for your warm invitation to this interesting authors work. I appreciate the authors of all their efforts. The study seems rigorous but it has many issues must be addressed before publication. Find my comments and suggestion hereunder.

Title

What is the importance of pronouncing “non-pregnant” ?

Introduction

- The authors mentioned that lactational amenorrhoea is a modern contraceptive method. Lactational amenorrhea method natural/or traditional method rather than modern method.
- The authors did not mention all the aims of family planning utilization.
- This section lacks consistency of ideas
- The authors did not describe the current figures of modern contraceptive utilization in Ethiopia.
- What efforts were made to boost the uptake of the modern contraceptive methods in the area?
- Line 126-131. You narrated general science. It would be better if you focused on your data analysis process.

Results

- Table 2 doesn’t clearly show statistically significant associations because it has no p-value.

Discussion

- This section is too shallow.

Major comment

- What makes your findings different from the 2019 Mini EDHS report?
- The authors need to address grammar and typo errors
- It seems authors focused on statistical analysis sections. The background and discussion sections should be written and interpreted by experts in the area.
