# Supplementary material for: Determinants of the use of modern contraceptives among women of reproductive age group in Ethiopia: A multi-level mixed effects analysis
Source: PLoS One. 2024 Jul 5;19(7):e0306635. doi: 10.1371/journal.pone.0306635 (PMC11226071; doi:10.1371/journal.pone.0306635)
Supplement: Attachment — Submitted filename: Point by point Authors response.docx [file pone.0306635.s003.docx]

Date: December 30, 2023

**Principal /Corresponding/ Author:** Gobena, Molalign Gualu (Assistant Professor of Statistics); Assosa University, Assosa, Ethiopia

Gmail:  molaligngualu[@gmail.com](mailto:maruzewdu68@gmail.com)

To: **Editor-in-Chief/Academic Editor**

**PLOS ONE Journal**

Dear Editor-in-Chief /Academic Editor,

Greetings!

Re: Manuscript Submission - [Determinants of the use of modern contraceptives among women of reproductive age group in Ethiopia: A multi-level mixed effects analysis]

We thank the reviewers and editors for their insightful feedback and recommendations on our manuscript, "Determinants of the use of modern contraceptives among women of reproductive age group in Ethiopia: A multi-level mixed effects analysis". We value the time and work you have taken to evaluate our work. We have addressed all of their points with significant revisions in response to their comments. We address every point raised by the reviewers in the section that follows.

We think that these changes have strengthened our manuscript considerably and taken care of all the reviewers' concerns. We hope that our updated work satisfies PLOS ONE Journal standards and is ready for publication. Thank you for taking a look at our updated manuscript.

Thank you once again for your time and attention to our submission.

Sincerely,

Molalign Gualu Gobena,

Principal/Corresponding Author

**Point by point author’s response to comments and questions**

| **No.** | **Comments and points raised** | **Author’s response** |
| --- | --- | --- |
| 1 | **Editor comments/efforts** | **Thank you!** |
|  | **Reviewer #1 comments/questions** | **Thank you!** |
| 1 | **In the title**: Why did you say non-pregnant? It’s better to update the title like, “Determinants of the use of modern contraceptives among women of reproductive age group in Ethiopia: A multi-level mixed effects Analysis”. Because, it’s obvious that pregnant women do not use contraceptive methods. | We have appreciated your suggestion and modified the title based on your comment. |
| 2 | **In the Abstract**: The authors applied a multi-level mixed effects Analysis to identify determinants of the use of modern contraceptives by simply mentioning multi-level mixed effects analysis without any appropriate reason. Multi-level modeling can be understood by a statistician but not the researchers with non-statistics background. Since the research does not focus on any statistical contribution rather then their application, the authors should mention at the beginning of the paper what and why multilevel modeling is necessary for such a study. | Thank you for your comment.  **Abstract:**  You are entirely correct. We recognize that the abstract needs to provide a clearer justification for the use of multilevel mixed-effects analysis (MLMEA). We have updated it to give a succinct justification of why multi-level modeling was selected as the best model for this investigation, emphasizing its benefit in taking community-level influences on individual contraceptive use into account. With this addition, the abstract will be more readable by researchers who are not statistical experts. |
| 3 | **In the Introduction**: First of all, the introduction lacks literature review on multi-level mixed effects analysis compared to the single level logistic regression analysis. I wonder the authors write "Even if several studies had examined and documented about the determinant factors associated with the use of modern contraception methods in Ethiopia, the impact of community level factors on it had received less consideration." Because, many studies have been done on the use of modern contraceptive among women of reproductive age group in Ethiopia using multilevel mixed effects analysis and 2019 EMDHS (The 2019 Ethiopian Mini Demographic and Health Survey) data. I don't know the importance of this study (your study). So, please state the gaps clearly authors have not stated clearly the gaps with many previous related studies in Ethiopia. Please state the holes clearly in this section.   - On page 2, lines 31& 32; you have said, “Among those 11 countries, Ethiopia is the best two countries in showing this steady increment.” What do you mean? Please correct it by, “Among those 11 countries, Ethiopia is one of the best two countries in showing this steady increment.” - On page 2, line 52; please correct the word “deference” by “difference”. In general, please enhance the quality of your paper by improving the English language (grammar, spelling and coherence of the sentences or paragraphs). | Thank you for your critical review and constructive comment!  **Introduction:**  **Literature Review on MLMEA**: We concur that it will be helpful to include a succinct review of the literature on MLMEA. We've included a special paragraph comparing and explaining why, because of its capacity to take into account both individual and community-level factors, MLMEA were chosen for our study.  **Gaps in Previous Research:** We apologize for the unclear articulation of research gaps. We have revised the introduction to clearly identify specific limitations in existing studies on modern contraceptive use in Ethiopia, particularly emphasizing the lack of focus on community-level influences. We have also clarified how our study addresses these gaps by employing MLMEA and analyzing relevant community-level variables.  **Sentences correction**: we have corrected the sentence based on your comment.  **Word correction**: We have rectified the typo "deference" to "difference".   - We have carefully proofread the whole document and made every effort to improve the English language throughout, as advised. |
| 4 | **In the results**:   - Please specify the level of significance at the beneath of table 2 and table 3. - Incorporate statistical findings, such as charts and maps, into your content. | This is also another constructive comment.  **Results:**  **Statistical Significance**: We have added a footnote showing the level of significance below Tables 2 and 3 to facilitate the interpretation of the results.  **Visualization**: We understand how important it is to include visual components like maps and charts. But, instead of it, we have used cross-tabulation to show descriptive statistics. |
|  | **Reviewer #2 comments/questions** | **Thank you!** |
| 1 | **Title**  What is the importance of pronouncing “non-pregnant”? | Thank you for question!  We have understood your question and changed the original title to "**Determinants of the use of modern contraceptives among women of reproductive age group in Ethiopia: A multi-level mixed effects analysis"**. |
| 2 | **Introduction**   - The authors mentioned that lactational amenorrhoea is a modern contraceptive method. Lactational amenorrhea method natural/or traditional method rather than modern method. - The authors did not mention all the aims of family planning utilization. - This section lacks consistency of ideas - The authors did not describe the current figures of modern contraceptive utilization in Ethiopia. - What efforts were made to boost the uptake of the modern contraceptive methods in the area? - Line 126-131. You narrated general science. It would be better if you focused on your data analysis process. | **Introduction:**  **Lactational Amenorrhea**: You are entirely correct. We regret that lactational amenorrhea was incorrectly categorized as a modern technique. As recommended, we have revised it.  **Aims of Family Planning:** We have recognized your point and expanded the introduction by including all key aims of contraceptive utilization.  **Consistency of Ideas**: We made an effort to improve the introduction in order to guarantee a coherent flow of concepts and remove abrupt changes. Moreover, we have logically grouped related information to improve the overall coherence.  **Modern Contraceptive Utilization Figures**: Please note that we have tried to provide the most up-to-date information (i.e. before 2021). Beyond this year the figures for modern contraceptive utilization in Ethiopia haven't been published.  **Boosting Contraceptive Uptake**: We have addressed your question by including relevant information.  **Data Analysis Process (Cross-Tabulation)**: We have revised the explanation under cross-tabulation section (in lines 126-131) by focusing on our data analysis process. |
| 3 | **Results**   - Table 2 doesn’t clearly show statistically significant associations because it has no p-value. | Thank you!  **Results:**  **Table 2**: We have appreciated your suggestion and added a column displaying p-values in the final fitted model (model 4) alongside confidence intervals. Please note that putting p-value for estimates in all models (model 1-4) consumes space. Instead of it, we have provided a confidence interval for each estimate in all models. It helps to identify statistically significant associations and give more comprehensive information than p-value. |
| 4 | **Discussion**   - This section is too shallow. | Thank you for your comment and suggestion. We have tried to make the discussion section to be deeper and more insightful. |
| 5 | **Major comment**   - What makes your findings different from the 2019 Mini EDHS report? - The authors need to address grammar and typo errors - It seems authors focused on statistical analysis sections. The background and discussion sections should be written and interpreted by experts in the area. | Thank you again for your major comment.  **Difference from the 2019 Mini EDHS report**:  The findings from this study differed in many ways from 2019 Mini EDHS report. Some of them can be expressed as follows:  **Findings from this study**   1) Based on Multi-level analysis 2) Provide both descriptive and Inferential statistics 3) Focus on reproductive aged women within Ethiopia 4) Have a policy implication by targeting this specific sub-population within Ethiopia 5) Provide the effect at community and individual level   **Findings from 2019 Mini EDHS report**   1) Potentially based on single-level analysis 2) Provide only descriptive statistics 3) Focus on the whole population within Ethiopia 4) Have a policy implication by targeting the whole population within Ethiopia 5) Provide the effect only at individual level   **Grammar and Typos**: After carefully going over the entire manuscript, we made an effort to ensure that there were no typos or grammatical errors.  **Expertise in Background and Discussion**: We fully agree that expertise in the relevant field is crucial for these sections. But, please notice that we ourselves are both statistician and public health expert. |

**Thank you!!!**
