# Supplementary material for: Determinants of the use of modern contraceptives among women of reproductive age group in Ethiopia: A multi-level mixed effects analysis
Source: PLoS One. 2024 Jul 5;19(7):e0306635. doi: 10.1371/journal.pone.0306635 (PMC11226071; doi:10.1371/journal.pone.0306635)
Supplement: Attachment — Submitted filename: Recommendation for authors.docx [file pone.0306635.s004.docx]

Recommendation for authors

Authors utilized the 2019 mini EDHS data of modern contraceptive methods. The proportion of modern contraceptive method utilization according to the mini EDHS was 41%. However, the authors found 30.4%. The same is true for each method. How this could be?

Moreover, how did the authors found those all individual and community levels variables? The 2019 mini EDHS data is limited a certain variables.

**Title**: it would be “Determinants of modern contraceptives utilization among reproductive age women in Ethiopia: A multi-level mixed effects Analysis”

Abstract: The authors concluded that young, wealthy, married, educated, fertile and, residents of City and Agrarian region reproductive-aged women in Ethiopia were relatively the highest users of modern contraception methods. I wonder how they reached to this finding. Do infertile women use any contraceptive method practically? Again the EDHS only include married women study, if so, how did you compare it with unmarried women?

Discussion

Line 323: Avoid etc. or mention all the rest findings.

Although authors found various significant variables, they did not discuss them all briefly.
