# Supplementary material for: Determinants of the use of modern contraceptives among women of reproductive age group in Ethiopia: A multi-level mixed effects analysis
Source: PLoS One. 2024 Jul 5;19(7):e0306635. doi: 10.1371/journal.pone.0306635 (PMC11226071; doi:10.1371/journal.pone.0306635)
Supplement: Attachment — Submitted filename: Point by point Authors response (2nd revision).docx [file pone.0306635.s005.docx]

Date: May 15, 2024

**Principal /Corresponding/ Author:** Gobena, Molalign Gualu (Assistant Professor of Statistics); Assosa University, Assosa, Ethiopia

Gmail:  molaligngualu[@gmail.com](mailto:maruzewdu68@gmail.com)

To: **Editor-in-Chief/Academic Editor**

**PLOS ONE Journal**

Dear Editor-in-Chief /Academic Editor,

Greetings!

Re: Manuscript Submission - [Determinants of the use of modern contraceptives among women of reproductive age group in Ethiopia: A multi-level mixed effects analysis]

We thank the reviewers and editors for their insightful feedback and recommendations on our manuscript, "Determinants of the use of modern contraceptives among women of reproductive age group in Ethiopia: A multi-level mixed effects analysis". We value the time and work you have taken to evaluate our work for the second time. We have addressed all of their points with revisions in response to their comments. We address every point raised by the reviewers in the section that follows.

We think that these changes have strengthened our manuscript considerably and taken care of all the reviewers' concerns. We hope that our updated work satisfies PLOS ONE Journal standards and is ready for publication. Thank you for taking a look at our updated manuscript.

Thank you once again for your time and attention to our submission.

Sincerely,

Molalign Gualu Gobena,

Principal/Corresponding Author

**Point by point author’s response to comments and questions**

**Reviewer #1 comments/questions with corresponding author’s response**

**General Comments**

The authors tried to address all the questions raised. But they have not included any graphs or charts in the result part.

**Author’s response:** Thank you for your continued review and feedback. We appreciate your suggestion to include graphs or charts in the results section.

We admit that charts and graphs are effective tools for presenting data because they are visual. But in this instance, we chose to employ cross-tabulations for the reasons listed below:

**Number of Variables**: Our study involved a relatively large number of socio-demographic factors potentially influencing modern contraceptive use. Presenting all these variables in separate graphs could be visually overwhelming and potentially obscure key findings. In addition, if we used graphs or charts in this manuscript, the number of figures becomes more than the number of figures allowed by the guideline of the journal.

**Detailed Information**: Cross-tabulations allow us to present detailed breakdowns of contraceptive use by each variable category (e.g., Age, education level, religion, household wealth index, etc…). This level of detail is crucial for understanding the specific relationships between these factors and contraceptive use.

**Clarity for Complex Relationships**: While graphs are excellent for showcasing trends, cross-tabulations are often better suited for illustrating complex interactions between multiple variables. This can be particularly relevant when analyzing data on social issues like contraceptive use.

**Reviewer #2 comments/questions with corresponding author’s response**

1) **In the Title;**

Title: it would be “Determinants of modern contraceptives utilization among reproductive age women in Ethiopia: A multi-level mixed effects Analysis”

**Author’s response:** I appreciate the title suggestion you made. We value your suggestions on how to make it more succinct and clear. The title "Determinants of modern contraceptives utilization among reproductive age women in Ethiopia: A multi-level mixed effects analysis" is the one that the reviewer preferred. We can understand that. We respectfully suggest retaining the original title, "Determinants of the use of modern contraceptives among women of reproductive age group in Ethiopia: A multi-level mixed effects analysis," even though it appropriately captures the essence of the study.

Here's our reasoning behind this preference:

**Clarity**: Our original title explicitly mentions "use" of modern contraceptives, which some readers might find easier to understand compared to "utilization."

**Target Audience**: Since the word "use" is frequently used in this context in Ethiopia, we think the original wording may be more understandable to policymakers and medical professionals there.

**Consistency**: The terminology used in the original title and the rest of the manuscript are in good alignment.

2) **In the Abstract**;

Abstract: The authors concluded that young, wealthy, married, educated, fertile and, residents of City and Agrarian region reproductive-aged women in Ethiopia were relatively the highest users of modern contraception methods. I wonder how they reached to this finding. Do infertile women use any contraceptive method practically? Again the EDHS only include married women study, if so, how did you compare it with unmarried women?

**Author’s response:** No. We apologize the confusion we made in the word “fertile” of this statement. We were used this word to mean women with more living children by considering the variable “living children”. We have revised it. In our study there is no a variable category name “fertile women” compared to infertile women. Please look the categories of the variable “living children” in the table 2. In this variable, the reference category is “No Child”. This is the reason that we were already tried to conclude by referring this reference category. Similarly, married women were not also compared with unmarried women. Rather it is compared with single women. This is because as it is a reference category of the variable “Marital status”. Please look it also from this table.

The EDHS does not include only married women. For detail, please look it on my response at result section.

3) **In the Methods;**

Moreover, how did the authors found those all individual and community levels variables?

**Author’s response:** We have considered variables measured at individual level as individual level variables whereas contextual variables that were measured at the higher level as a community level variables.

The 2019 mini EDHS data is limited a certain variables.

**Author’s response:** Yes, but, we have used the available variable which are of our variables of interest in the study.

4) **In the Results**;

Authors utilized the 2019 mini EDHS data of modern contraceptive methods. The proportion of modern contraceptive method utilization according to the mini EDHS was 41%. However, the authors found 30.4%. The same is true for each method. How this could be?

**Author’s response:** Please note that the 2019 Mini EDHS does not only include married women. It includes all reproductive aged women (15-49). Please look it again the report on page 37 under the title “5.1 CONTRACEPTIVE KNOWLEDGE AND USE”. Please give attention the two shaded sentences under this title. Hence, the findings under this title make use of “All women age 15-49 and currently married women aged 15-49” as a sample. I think your comment on percentage of modern method is based on the report sentence “Many currently married women use a modern method (41%), while only 1% use a traditional method” on page 38. This statistics is not applied for all reproductive aged women. This is only applied only for currently married women. To find the full finding, please look the Table 5.3 on page 43. In this table the percentage of modern contraceptive use among all reproductive aged women are 28.1%. But, in our study it is 30.4%. The possible differences may be we used only 8196 reproductive aged women where as they used 8885 reproductive aged women as a sample. This implies we remove 689 cases in our analysis. This is because we were made a careful multicomponent sexual activity profile (MSAP) of all reproductive-aged women consideration to identify sexually active women. As a result, we have identified 8196 reproductive-aged women as sexually active women.

5) **In the Discussion**;

Line 323: Avoid etc. or mention all the rest findings.

Although authors found various significant variables, they did not discuss them all briefly.

**Author’s response:** Thank you for your continued review and feedback. We appreciate you highlighting the importance of comprehensive discussion in the manuscript. In our first revision, we felt as we have discussed all significant variables found in the study, albeit briefly. We understand that conciseness is important, but we also want to ensure all relevant findings are addressed. In line 323, we have avoided etc.
