# Supplementary material for: Determinants of the use of modern contraceptives among women of reproductive age group in Ethiopia: A multi-level mixed effects analysis
Source: PLoS One. 2024 Jul 5;19(7):e0306635. doi: 10.1371/journal.pone.0306635 (PMC11226071; doi:10.1371/journal.pone.0306635)
Supplement: Attachment — Submitted filename: Reviewer Comments to Authors.docx [file pone.0306635.s001.docx]

**Reviewer Comments to authors**:

The authors of the paper, " Determinants of the use of modern contraceptives among non-pregnant reproductive-aged women in Ethiopia: A multi-level mixed effects Analysis“, structured the paper nicely covering both statistical theories on a multi-level mixed effects Analysis and its application to the use of modern contraceptives among non-pregnant reproductive-aged women in Ethiopia. I found the paper contributes little to either statistical modeling or the use of modern contraceptives literature. I found some issues discussed below:

1. **In the title**: Why did you say non-pregnant? It’s better to update the title like, “Determinants of the use of modern contraceptives among women of reproductive age group in Ethiopia: A multi-level mixed effects Analysis”. Because, it’s obvious that pregnant women do not use contraceptive methods.
2. **In the Abstract**: The authors applied a multi-level mixed effects Analysis to identify determinants of the use of modern contraceptives by simply mentioning multi-level mixed effects analysis without any appropriate reason. Multi-level modeling can be understood by a statistician but not the researchers with non-statistics background. Since the research does not focus on any statistical contribution rather then their application, the authors should mention at the beginning of the paper what and why multilevel modeling is necessary for such a study.
3. **In the Introduction**: First of all, the introduction lacks literature review on multi-level mixed effects analysis compared to the single level logistic regression analysis. I wonder the authors write "Even if several studies had examined and documented about the determinant factors associated with the use of modern contraception methods in Ethiopia, the impact of community level factors on it had received less consideration." Because, many studies have been done on the use of modern contraceptive among women of reproductive age group in Ethiopia using multilevel mixed effects analysis and 2019 EMDHS (The 2019 Ethiopian Mini Demographic and Health Survey) data. I don't know the importance of this study (your study). So, please state the gaps clearly authors have not stated clearly the gaps with many previous related studies in Ethiopia. Please state the holes clearly in this section.

- On page 2, lines 31& 32; you have said, “Among those 11 countries, Ethiopia is the best two countries in showing this steady increment.” What do you mean? Please correct it by, “Among those 11 countries, Ethiopia is one of the best two countries in showing this steady increment.”
- On page 2, line 52; please correct the word “deference” by “difference”. In general, please enhance the quality of your paper by improving the English language (grammar, spelling and coherence of the sentences or paragraphs).

4. **In the results**:
   -     Please specify the level of significance at the beneath of table 2 and table 3.

- Incorporate statistical findings, such as charts and maps, into your content.
